# Supplementary material for: Assessment of low-vault cases with an implantable collamer lens
Source: PLoS One. 2020 Nov 4;15(11):e0241814. doi: 10.1371/journal.pone.0241814 (PMC7641440; doi:10.1371/journal.pone.0241814)
Supplement: Attachment — Submitted filename: Kato_S_Response to Reviewers.docx [file pone.0241814.s002.docx]

October 18,2020

To,

Editorial Board

*PLOS ONE*

Dear Editors:

On behalf of my co-authors, I thank you for the opportunity to revise and resubmit our manuscript titled, “Assessment of low-vault cases with an implantable collamer lens.” We also thank the reviewers for their insightful comments, suggestions, and questions, which we believe have helped us improve our article considerably. Several valid points were raised by the reviewers, and after careful consideration, we have made the requisite revisions to the manuscript. Below, you will find our point-by-point responses to the reviewers’ comments, questions, and concerns (in red text). Our revised manuscript is attached as a separate document, with the corresponding changes highlighted in red text.

We sincerely hope that our responses and revisions have adequately addressed the reviewers’ concerns, and that our manuscript is now suitable for publication in *PLOS ONE.*

Once again, we thank you for providing us the opportunity to publish our manuscript in your journal.

Sincerely,

Sayaka Kato, CO, Eye Center, Sanno Hospital, 8-10-16 Akasaka, Minato-ku, Tokyo 107-0052, Japan.

Telephone: +81-3-3402-3151;

Fax: +81-3-3403-3652;

E-mail: sayaka331010843@yahoo.co.jp

**We would like to thank all the reviewers for critically appraising our work, and offering insightful comments and constructive suggestions to improve our article.**

Reviewer #1:

The authors focused on low vault cases after implantable collamer lens implantation surgery and evaluated the efficacy, safety, predictability, and complications.

It has been pointed out that the low vault has a risk of cataract formation and toric ICL rotation. For this reason, it is important to collect and analyze only low vault cases, which seems to be the novelty of this paper.

#1: In the Introduction and Discussion section, the low vault is referred to as a case of less than 250 µm. However, the Methods section doesn't show at what point in time or how many vaults were defined as low vaults.

**Response:**

Thank you for pointing this out, we added the text to the manuscript.

**(Page 4, line 64)**: “low vault (<250 μm)”

#2: Citing Lee's paper in the Discussion section, the authors state that the vault is not related to the toric ICL rotation, but some studies have reported that the vault is correlated (Sheng XL, Rong WN, Jia Q, et al. Outcomes and possible risk factors associated with axis alignment and rotational stability after implantation of the Toric implantable collamer lens for high myopic astigmatism. Int J Ophthalmol. 2012;5(4):459-465.). This issue should be mentioned in detail in the discussion section.

**Response:**

Thank you for pointing this out, we added the text to the manuscript.

**(Page 12, lines 237-238)**: “Sheng et al. [11] reported that a lower vault was correlated with an increased risk of Toric ICL rotation. On the other hand,”

#3: Authors measure the central vault, but in myopic ICL, the peripheral vault is closest to the lens due to the shape of the lens. The limitations of measuring only the central vault should be mentioned in the discussion.

**Response:** discussion

Thank you for pointing this out, we added the text to the manuscript.

**(Page 11, lines 204-208)**: “Myopic eyes with ICLs are thicker at the periphery than in the center. However, currently, it is challenging to measure the peripheral vault using anterior segment OCT, as the iris interferes with the measurement. The addition of a hole in the ICL allows the circulation of the aqueous humor and reduces complications, even in eyes with a low vault. Therefore, vault evaluation of this study was performed only for the center of the lens.”

#4: It has been reported that vault measurements dynamically change in examination environment such as brightness. CASIA measurement conditions should be added in Methods section.

**Response:**

Thank you for pointing this out, we added the text to the manuscript.

**(Page 5, line 78)**: “under photopic conditions (background illumination = 400 lx).”

Reviewer #2:

Authors specifically examined the cases with low vault after Hole ICL, then reported the safety and efficacy of Hole ICL only just for one year. This research is clinically relevant, but I actually don't know if the sample volume that authors examined in this clinical research is sufficient to come to the conclusion. Also, authors observed the cases just for 1 year. The safety of the surgery would not be able to be seen in one year only.

#1: Authors mentioned that the aim in this research was to examine the clinical results of visual outcomes and long-term complications. But just 1-year follow-up is not long term. Authors should change the purpose of this research.

**Response:**

Thank you for pointing this out, we modified the text in the manuscript.

**(Page 2, lines 15)**: “over a one-year follow-up period.”

#2: Authors examined 16 patients, 25 cases who underwent Hole ICL surgery, then exhibited low vault. How frequently have the authors seen the cases with low vault after Hole ICL surgery ? Readers may not understand how commonly it is observed after Hole ICL. Authors should address the background information on it in either Introduction or Discussion section.

**Response:**

Thank you for pointing this out, we added the text to the manuscript.

**(Page 11, lines 202-204)**: “Kojima et al. [15] reported that, one year postoperatively, 13.9% of patients had a low vault (≤250 μm), 72.2% had a moderate vault (250­–750 μm), and 13.9% had a high vault (≥750 μm).”

#3: Abstract, Line 23 “implantable collamer lens size was 12.1, 12.6, and 13.2 mm for…”

Please create a large capital letter at the beginning of a paragraph.

**Response:**

Thank you for pointing this out, we modified the text in the manuscript.

**(Page 2, line 23)**: “Implantable collamer lens”

#4: Citation 8, This is a meta-analysis and review paper. Authors should cite the original paper.

**Response:**

Thank you for pointing this out, we modified the text as follows.

**(Page 3, lines 42-44)**:

“The central hole improves aqueous humor circulation, preventing many of these complications ~~[8]~~, without the need for peripheral iridectomy, as is required when utilizing the ICL V4 [6,8,9].“

**(Page 3, lines 48-49)**:

“Shimizu et al. [8], utilizing Hole ICL, reported a postoperative cataract rate of 0% was reported at a 5-year follow-up;”

**(Page 12, lines 227-229)**:

“In previous studies, anterior subcapsular opacities occurred after conventional ICL in 1.1–5.9% of cases by 5 years after the operation, and 0–1.8% of total cases required cataract surgery [~~8~~8,20-22]. “

**(Page 12, lines 230-231)**:

“Conversely, none of the cases receiving Hole ICL surgery exhibited anterior subcapsular opacities or visually significant cataracts, irrespective of vault [~~8~~ 8]”

#5: ICL is a posterior chamber lens, but author should have a discussion regarding postoperative ECD in Discussion. ECD loss is one of the most serious complications.

ICLは後眼房レンズですが、著者は術後ECDに関する議論を議論する必要があります。 ECDの喪失は、最も深刻な合併症の1つです。

**Response:**

Thank you for pointing this out, we added the text to the manuscript.

**(Page 12, lines 233-236)**:

“Goukon et al. [23] reported that the mean central ECD loss was 0.3%, 2 years after Hole ICL. Although no statistically significant decrease in ECD was observed in our study, the sample size was small and the follow-up was only 1 year after surgery; future studies with long-term follow-up periods are required.”
